# Predictive model for bacterial late-onset neonatal sepsis in a tertiary care hospital in Thailand

**DOI:** 10.1186/s12879-020-4875-5

**Published:** 2020-02-18

**Authors:** Dominicus Husada, Pornthep Chanthavanich, Uraiwan Chotigeat, Piyarat Sunttarattiwong, Chukiat Sirivichayakul, Krisana Pengsaa, Watcharee Chokejindachai, Jaranit Kaewkungwal

**Affiliations:** 1 Department of Child Health, School of Medicine Airlangga University/Dr. Soetomo Hospital, Surabaya, 60286 Indonesia; 2 Faculty of Tropical Medicine, Mahidol University, Bangkok, Thailand; 3 Queen Sirikit National Institute of Child Health, Bangkok, Thailand

**Keywords:** Predictive model, Bacterial late-onset neonatal sepsis, Scoring system, Thailand

## Abstract

**Background:**

Early diagnosis of neonatal sepsis is essential to prevent severe complications and avoid unnecessary use of antibiotics. The mortality of neonatal sepsis is over 18%in many countries. This study aimed to develop a predictive model for the diagnosis of bacterial late-onset neonatal sepsis.

**Methods:**

A case-control study was conducted at Queen Sirikit National Institute of Child Health, Bangkok, Thailand. Data were derived from the medical records of 52 sepsis cases and 156 non-sepsis controls. Only proven bacterial neonatal sepsis cases were included in the sepsis group. The non-sepsis group consisted of neonates without any infection. Potential predictors consisted of risk factors, clinical conditions, laboratory data, and treatment modalities. The model was developed based on multiple logistic regression analysis.

**Results:**

The incidence of late proven neonatal sepsis was 1.46%. The model had 6 significant variables: poor feeding, abnormal heart rate (outside the range 100–180 x/min), abnormal temperature (outside the range 36^o^-37.9 °C), abnormal oxygen saturation, abnormal leucocytes (according to Manroe’s criteria by age), and abnormal pH (outside the range 7.27–7.45). The area below the Receiver Operating Characteristics (ROC) curve was 95.5%. The score had a sensitivity of 88.5% and specificity of 90.4%.

**Conclusion:**

A predictive model and a scoring system were developed for proven bacterial late-onset neonatal sepsis. This simpler tool is expected to somewhat replace microbiological culture, especially in resource-limited settings.

## Background

Neonatal sepsis is a global challenge causing high morbidity and mortality among newborns [1–4]. The global infant mortality rate in 2014 was 29 per 1000 live births—the common cause being infection [5]. Neonatal sepsis accounted for 1.4 million neonatal deaths or around 40% of total lives lost, annually [6] About 99% of neonatal deaths occur in low and middle-income countries (LMIC) and approximately 62% occurred during the first 3 days of life [7].

The exact data of neonatal sepsis in the LMIC is limited [8–11]. Two studies from Nigeria showed a prevalence rate of 47.2 and 21.8% [12, 13]. A study from Indonesia found 46.6% prevalence [14]. In Thailand, two decades ago, the prevalence of late-onset neonatal sepsis at Siriraj Hospital, the largest hospital in the country, was 0.05/1000 live births [15]. Ramathibodhi Hospital in Bangkokalso recorded almost similar prevalence [16]. Another study from 2012, involving 4 countries, including Thailand, found a prevalence of 21.22 per 1000 admissions [17].

Neonatal sepsis is defined as a clinical syndrome of bacteremia with systemic signs and symptoms of infection in the first 4 weeks of life [18]. Although various organisms can cause neonatal sepsis, the focus of this study was bacterial sepsis. Bacteria are the most common cause of neonatal sepsis in the world [2, 4, 5].

There are two types of neonatal sepsis, early- and late-onset. There is little consensus about applicable age limits in literature [19]. Usually, the age limit defined for early-onset sepsis varies from 3 to 7 days [1, 20]. Some clinicians and researchers use 7 days as the limit [17, 19, 21, 22]. Late-onset sepsis is usually caused by organisms acquired after delivery and considered as nosocomial community-acquired infection [17, 22].

Many factors contribute to newborns’ susceptibility to sepsis. The common risk factors are maternal, neonatal, and other conditions that predispose infants to infections, such as invasive procedures [19, 22–25]. Neonates born early or with very low birth weight are highly likely to contract sepsis [2, 26, 27].

Early diagnosis of sepsis improves survival and functional outcome [28, 29]. The other benefit of an early and correct diagnosis is related to antibiotic consumption. A five-year study in Poland revealed the reduction of antibiotic usage [30]. Overuse of antibiotics causes resistance problems worldwide [31].

The detection of neonatal sepsis is difficult due to the non-specific clinical signs and symptoms and the relative diagnostic inaccuracy of the available parameters or biomarkers [32]. Many of non-infectious syndromes have initial clinical presentations similar to severe infections [33] The gold standard for diagnosis of systemic bacterial infection is the isolation of pathogens, usually from peripheral blood. Unfortunately, the sensitivity of this method is low. Thus, the diagnosis of sepsis cannot be excluded even when the results are negative [34, 35]. When the cultures are negative, but the infant manifests signs consistent with an infection, it may be assumed that they have clinical sepsis [3].

The clinical prediction rule (or predictive model, probability assessment, decision rule, risk score) [36] is a decision-making tool for clinicians with three or more variables obtained from the history, physical examination, and simple diagnostic tests. They are derived from the data collected directly from patients [36–38]. They provide powerful tools to improve clinical decision making [39].

Predictive models quantify the relative importance of individual clinical indicators for evaluating the risk of an adverse outcome for an individual patient [40]. These models attempt to formally test, simplify, and increase the accuracy of a clinician’s diagnostic and prognostic assessment and are most likely to be useful in situations where decision making is complex, the clinical stakes are high, or there are opportunities to achieve cost savings without compromising patient care [36, 41, 42]. This study aimed to develop a predictive model for the diagnosis of late-onset neonatal sepsis. The model, expectedly, helps clinicians determine the infection status of the neonates without waiting for the microbiology facility.

## Methods

### Study design and site

This case-control study was performed at Queen Sirikit National Institute of Child Health (QSNICH), Bangkok, Thailand. It has 3 neonatal sick wards, including 1 neonatal intensive care unit (NICU). The initial dataset was compiled from three year periods of the medical record in 2005–2007 and then recalculated in 2014. The need in specific areas was considered, especially in many parts of low- and middle-income countries, including South East Asia. Many areas are immensely burdened by neonatal sepsis patients and require simple tools to overcome the difficulty with the microbiology culture facilities.

### Samples

Neonates diagnosed with sepsis were included in the case group. Late-onset neonatal sepsis was defined as sepsis at 7 days or more. The inclusion criteria were: age < 28 days on admission, sepsis as the final diagnosis (either main diagnosis or additional), and at least one positive laboratory test for a bacterial pathogen (It could bepositive bacterial culture result/polymerase chain reaction (PCR)/gram-staining/latex agglutination tests/antigen-antibody detection for bacteria). The hospital used BacTec (Becton Dickinson Microbiological System, Maryland) for bacterial culture. All the patients with severe congenital malformation that underwent surgery before the diagnosis of sepsis or were admitted for less than 6 h in the hospital were excluded. Inclusion criteria for the control group were: age < 28 days on admission, a final diagnosis other than sepsis, admitted in range of 20 days before or after the comparing sepsis patient, except for the NICU where the time range was expanded to the same year, hospitalized in the same ward with the comparing case, and at least 7 days old on the day of data taken. Thus, each case had 3 controls.

### Definitions

Neonates: an infant who is less than four weeks old.

Late-onset neonatal sepsis: sepsis diagnosed among neonates at the age of 7 days or more [24, 26, 28].

Clinical sepsis: sepsis in which blood cultures are not performed, not detected, or for which the physician institutes treatment for sepsis. Clinical sepsis patients were not used in this study.

### Data collection and management

The dependent variable in this study was proven sepsis. The independent variables had 4 categories: risk factors (basic/demographic data, maternal history: antepartum, intrapartum, and postpartum), clinical manifestations, laboratory findings, and treatment modalities. Initially, 144 variables were considered.

Data collection began by obtaining the list of neonatal patients from the medical record office. The three-year data were compiled and divided into three groups: (a) sepsis with positive bacterial culture result/PCR/gram-staining/latex agglutination tests/antigen-antibody detection for bacteria, (b) clinical sepsis, but without definite specific results as mentioned in the group (a), and (c) non-sepsis. Patients in the group (b) were not included in this study. Group (a) was identified using the International Classification of Diseases (ICD)-10 code of P360 to P368; meanwhile, the ICD-10 code for group (b) was P369.

While selecting the “sepsis group,” the data from culture result records in the neonatal ward was also searched to increase the number of subjects. All medical records of the sepsis group were checked to ensure the fulfillment of inclusion criteria. Subsequently, data from medical records were transferred to case record forms. For clinical and laboratory examinations, the data used were the worst result (could be highest or lowest) in the range of 24 h before or after the diagnosis of sepsis. If such data were not available, the most recent previous data were chosen. The name, address, and hospital number of the patients were not recorded as case records. The hospital numbers were only written in the master log record.

After obtaining all data for the sepsis group, the patients were divided according to the date of admission and the ward/site in the hospital. The control group was selected based on this division and the master medical record list. Controls were matched to the sepsis group based on: (a) date of admission (in the range of 20 days before or after the cases) and (b) hospitalized in the same ward (9, 10, or NICU) with the comparing sepsis patient, and (c) at least 7 days of age. The amount of control: sepsis patients were 3:1. The medical records of patients in the control group were checked to ensure the fulfillment of inclusion criteria. Data from medical records were then transferred to the case record forms. For the control group, the data used were the worst after 7 days of age. Therefore, the records of clinical conditions and laboratory results were observed daily. All the patients in the control group were not diagnosed with sepsis before the data were taken. All data from case record form were transferred to Statistical Package for the Social Sciences (SPSS) database, and data accuracy was rechecked after completing every single record form.

### Data analysis

Once the data were available, descriptive, univariate (with t-test, Mann Whitney U, or Chi-Square tests)—as appropriate, and multivariable analysis with multiple logistic regression and calculation of diagnostic test aspects (sensitivity, specificity, positive predictive value (PPV), negative predictive value (NPV), likelihood ratio (LR), and receiver operating characteristics (ROC) Curve) were performed. All univariate analysis used two-tailed *p*-value < 0.05. Multivariable analysis used p-value < 0.1. The software used for data analysis was SPSS Version 11.5 (SPSS Inc., Chicago, IL).

The first step of the analytical process was evaluating missing data. Variables with too many incomplete data were not used. For the remaining variables, the missing data were replaced by the imputation method. For the control group, the mean of normal value (based on the literature) were considered. The second step was descriptive analysis. This was done by finding the frequency distributions, mean + standard deviation (SD), and median (and range).

The third step was the univariate tests, which were done to compare 2 groups: sepsis and non-sepsis. The tests used for comparison were t-test, Mann Whitney U, and chi-square test, depending on the type of data. Variables with *p* > 0.1 were excluded. The variables with *p* < 0.1 proceeded to the next step. The fourth step was the selection of remaining variables based on clinical consideration, collinearities, and similarities. The fifth step was the multivariate analysis by multiple logistic regression using the “enter” method. The considerations for the final decision were: number of variables, ease of usage, clinical judgment, performances, and results from several other studies as the comparison. This process resulted in the final equation. The sixth step calculated the sensitivity, specificity, PPV, NPV, LR, and the ROC curve on certain cutoff values of the final equation (or model). In the final, seventh step, the equation was transformed into a scoring system for practical purposes. This scoring system was developed based on the coefficients of each variable in the equation. Some proposed score systems (varying in the process of rounding coefficients) were tried and the best results were selected based on the ROC curve.

### Ethical approval

Ethical approval for this study was obtained from two Ethical Committees—The Faculty of Tropical Medicine, Mahidol University, and Queen Sirikit National Institute of Child Health, Bangkok.

## Results

### Searching for medical records

**Fig. 1:**
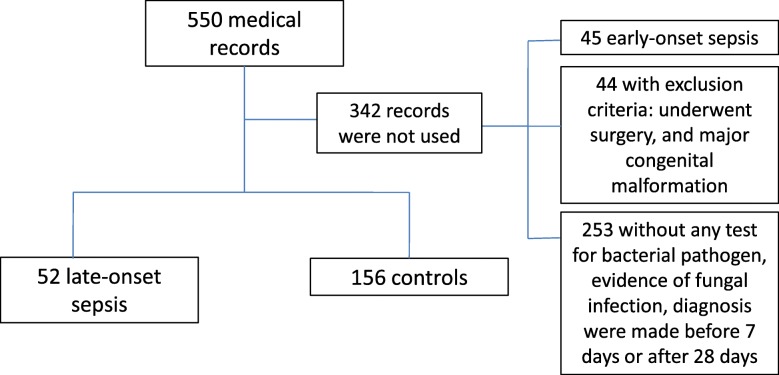
The Medical Records Searching Results

The study explored 550 medical records from the Medical Record Unit Queen Sirikit National Institute of Child Health (QSNICH), Bangkok. Finally, there were 52 neonates with late-onset sepsis and 156 controls. Forty-five neonates with early-onset sepsis and the other 297 participants were not included because they did not fulfil the inclusion criteria or because of the exclusion criteria. Figure 1 illustrates the results of medical records searching.

### Patient characteristics

For 3 years, there were 3557 neonatal patients admitted in QSNICH. This study used 11% of the total neonatal patients. Table 1 lists some baseline characteristics of the studied neonates. Most of the neonatal patients in QSNICH were male, weighted between 2500 and 4000 g, and were admitted in the first 24 h of their life. The overall incidence of proven neonatal sepsis at Queen Sirikit Institute of Child (QSNICH) Bangkok was 2.7% (denominator: all neonatal patients in QSNICH). The incidence of proven late-onset neonatal sepsis (LOS) was 1.46%.

**Table 1:** Baseline Characteristics of Neonatal Patients in the Study

	Case	Control	*p* VALUE
• Median age at sepsis/date of data taken for control (days)	15 (8–28)*	10 (8–28)*	**< 0.001**^$^
• Median length of hospital stay before sepsis/date of data taken for control (days)	11 (0–27)*	7 (0–25)*	**< 0.001**^$^
• Median gestational age (weeks)	32 (26–41)*	35 (26–42)*	**0.041**^$^
• Mean (SEM^#^) of mother age (years)	25.77 (0.98)	26.99 (0.46)	0.085
• Maximum space from previous child (years)	20	10	**< 0.001**^$^
• Maximum length of hospital stay (days)	94	114	**< 0.001**^**$**^

Note: *in the brackets are the minimum-maximum values

^$^significant

^#^Standard Error of the Mean

The most common diagnosis among the control group was hyperbilirubinemia (79%). The other diagnosis was asphyxia, apnea of prematurity, and respiratory disease syndrome.

### Microbiology and antibiotic

There were 52 neonatal patients who showed positive culture results from the blood. Three patients also had positive gram stain from cerebrospinal fluid (CSF), and 1 had a positive latex agglutination test from CSF. All those gram stain and latex agglutination test results were comparable with the hemoculture. Among the control group, 2 patients had positive hemoculture for *Coagulase-negative Staphylococcus* (CONS) and 1 had a positive enzyme-linked immunosorbent assay (ELISA) test for dengue infection. However, the data from these 2 patients with CONS were taken before the culture procedure. The most common bacteria were *Klebsiella pneumoniae*, CONS, and *Enterobacter* spp. Ampicillin was used as a single or combination drug for 78% of the septic neonates in this study.

### Comparison of outcome

Most of the patients (53.3%) developed sepsis during the age of 15–28 days. These differ from the control group (*p* < 0.001). The patients who had sepsis had a significantly higher mortality rate and a longer hospitalization compared to the control group. Table 2 lists the comparison between the outcome, age, sex, and the duration of hospitalization.

**Table 2:** Comparison of Outcome, Age, Length of Hospital Stay, Sex, and Referral Source between Sepsis and Non Sepsis Patients

Variables	Case (*n* (%))	Control (*n* (%))	*p* value
Outcome			**0.001**^$^
• Alive	44 (84.6)	153 (98.1)	
• Death	8 (15.4)	3 (1.9)	
Age on sepsis (/date of data taken for control)			< 0.001*
• 8–14 days	22 (42.3)	120 (76.9)	
• 15–28 days	30 (57.7)	36 (23.1)	
Length of hospital stay before sepsis (/date of data taken for control)			< 0.001*
• 0–24 h	13 (25)	62 (39.7)	
• 2–3 days	1 (2)	6 (3.9)	
• 4–7 days	5 (9.6)	43 (27.6)	
• 8–14 days	14 (26.9)	39 (24.9)	
• > 14 days	19 (36.5)	6 (3.9)	
Total length of hospital stay			< 0.001*
• 0–24 h	1 (2)	3 (1.9)	
• 2–3 days	0	23 (14.7)	
• 4–7 days	0	20 (12.8)	
• > 7 days	–	–	
• 8–14 days	5 (9.6)	31 (19.9)	
• 15–30 days	14 (26.9)	41 (26.3)	
• > 30 days	32 (61.5)	38 (24.4)	
Sex			0.470^$^
• Male	26 (50)	87 (55.8)	
• Female	26 (50)	69 (44.2)	
Referred from			0.606^$^
• Rajvithi hospital	25 (48.1)	75 (48.1)	
• Other hospital	15 (28.8)	36 (23.1)	
• Others	12 (23.1)	45 (28.8)	

Notes: ^$^ = Chi-square test; * = Mann-Whitney U Test

### Comparison of risk factors

The odds ratio (OR) regarding the risk factors for sepsis are listed in Table 3. Over 50% of neonatal sepsis patients were born from high-risk pregnancies, compared to only 35% in the control group. Most of their mothers were aged between 15 and 30 years, and were employed as laborers or were unemployed and lived in the slum area. Most of them were educated until elementary or high school. 51.9% mothers were given a steroid injection before birth which protected the neonates. Premature rupture of the membranes was not significantly different from the control group in the sepsis group. Only 6 mothers from this study had chorioamnionitis. Preeclampsia was the most common complication in the pregnancies, (9 cases). The majority of all neonates had good Apgar score either at the first or fifty minutes. The highest odds ratio for risk factors was found for the duration of the hospitalization (4.284), intracranial hemorrhage (3.419), high-risk pregnancies (2.727), and resuscitation of the neonates (2.060).

**Table 3:** Odds Ratio for Risk Factors between Sepsis and Non-Sepsis Patients

Variables	OR	95% CI	*p* value
Demographic			
• Sex (male: female)	1.3	0.672–2.364	0.47^$^
• Length of hospital stay before sepsis (< 7:> 7 days)	**4.3**	2.209–8.308	< 0.001*
Antepartum history			
• Race (Thai: non-Thai)	1.5	0.270–8.550	0.327^$^
• Smoking (Yes: No)	3.0	0.187–49.472	0.438^$^
• Chronic disease of mother (Yes: No)	1.0	0.374–2.671	0.218^$^
• High risk pregnancy (Yes: No)	**2.7**	1.434–5.188	0.002^$^
• Long term drug user (Yes: No)	1.0	0.196–5.114	1.00^$^
Intrapartum history			
• Mode of delivery I (Caesarian section: non-operative)	1.0	0.53–1.888	0.561^$^
• Mode of delivery II (Non-spontaneous: spontaneous)	1.0	0.547–1.926	0.662^$^
• Premature rupture of membrane (Yes: No)	2.2	0.857–5.815	0.105^$^
• Chorioamnionitis (Yes: No)	1.1	0.110–10.680	1.00^$^
• Smelly amniotic fluid (Yes: No)	1.6	0.145–18.742	0.555^$^
• Fever of mother (Yes: No)	2.1	0.345–13.093	0.597^$^
• Steroid injection before birth (Yes: No)	**0.4**	0.203–0.737	0.003^$^
• Antibiotics before birth (Yes: No)	0.8	0.39–1.467	0.279^$^
• Prematurity (20–36 weeks: 37<)	0.7	0.374–1.330	0.542*
• Intracranial hemorrhage (Yes: No)	**3.4**	1.277–9.149	0.019^$^
• Resuscitation (Yes: No)	**2.1**	1.084–3.916	0.026^$^
Postpartum history			
• Icterus after birth (Yes: No)	1.3	0.428–3.812	0.770^$^
• Seizure (Yes: No)	1.5	0.134–17.000	1.00^$^
• Breastfed (Yes: No)	1.6	0.781–3.128	0.205^$^

Notes: ^$^ = Chi-square test; * = Mann-Whitney U test

### Comparison of cclinical condition, laboratory data, and treatment modalities

The odds ratio (OR) of clinical conditions and laboratory data for sepsis are listed in Table 4. The highest OR for clinical condition, laboratory data, and treatment modalities were abnormal heart rate (40.765), abnormal CSF glucose (24.771), and central or umbilical catheter (6.622), respectively. All data of vascular catheter and total parenteral nutrition (TPN) were taken before the sepsis diagnosis.

**Table 4:** Odds Ratio for Clinical Conditions, Laboratory Data, and Treatment Modalities between Sepsis and Non-Sepsis Patients

Variables	OR	95% CI	*p* value^$^
Clinical Condition			
• Lethargy (Yes: No)	**15.9**	7.385–33.737	< 0.001
• Poor feeding (Yes: No)	**18.4**	8.477–39.927	< 0.001
• Cyanosis (Yes: No)	1.4	0.463–4.243	0.554
• Icterus in hospital (Yes: No)	**0.2**	0.087–0.620	0.002
• Abnormal heart rate (Yes: No)	**40.8**	9.029–184.041	< 0.001
• Abnormal temperature (Yes: No)	**24.8**	6.880–89.195	< 0.001
• Apnea episode (Yes: No)	**17.0**	4.616–62.608	< 0.001
• Respiratory insufficiency (Yes: No)	**11.0**	5.090–23.931	< 0.001
• Hypoxemia (Yes: No)	**14.2**	6.273–32.309	< 0.001
Laboratory Data			
• Anemia (Yes: No)	**5.1**	2.593–10.067	< 0.001
• Abnormal leucocytes (Manroe’s) (Yes: No)	**17.4**	6.056–49.921	< 0.001
• Thrombocytopenia (Yes:No)	**9.5**	4.260–21.264	< 0.001
• Abnormal pH (Yes: No)	**18.3**	8.437–39.577	< 0.001
• Abnormal base excess (Yes: No)	**10.5**	4.597–23.778	< 0.001
• Abnormal potassium (Yes:No)	**4.1**	2.108–7.869	< 0.001
• Abnormal sodium (Yes: No)	**4.4**	1.764–10.872	0.001
• Abnormal BUN* (Yes: No)	**10.1**	3.681–27.095	< 0.001
• Abnormal CSF^#^ WBC^&^ (Y:N)	**14.7**	5.068–42.456	< 0.001
• Abnormal CSF glucose (Y:N)	**24.8**	6.880–89.195	< 0.001
• Abnormal CSF protein (Y:N)	**11.1**	3.774–32.806	< 0.001
Treatment Modalities			
• Central/Umbilical catheter (Yes: No)	**6.6**	3.332–13.161	< 0.001
• Total parenteral nutrition (Yes: No)	**5.8**	2.810–11.927	< 0.001

Notes: ^$^Chi-Square test

*Blood urea nitrogen

^#^Cerebrospinal fluid

^&^White blood cell

### The equation and probability of proven sepsis

Multiple logistic regression produced the following final result: Y = (2.398 * poor feeding) + (3.087 * abnormal heart rate) + (3.995 * abnormal temperature) + (1.387 * abnormal oxygen saturation) + (1.786 * abnormal leucocytes) + (2.479 * abnormal pH) – 4.328. The formula for the probability (P) was P = {EXP (Y)} / {1 + EXP (Y)}.

The coding for this equation is listed in the Supplementary Material (Additional file 1). There were 6 variables in the final regression equation—4 from the clinical condition and 2 from laboratory data. The result of the regression equation was placed in the exponential equation to calculate the probability. Probability implies the probability to have proven sepsis in this equation and is expressed as a percentage. Table 5 lists the odds ratio and adjusted odds for all variables in the equations.

**Table 5:** Odds Ratio and Adjusted Odds of Variables Used in The Final Equation

Variables	ODDS RATIO	95% CI		Adjustodds	95% CI		p value^@^
		Lower	Upper		Lower	Upper	
Clinical							
Poor feeding	18.397	8.477	39.927	10.996	3.201	37.776	< 0.001
Abnormal heart rate	40.765	9.029	184.041	21.920	2.235	215.020	0.008
Abnormal temperature	24.771	6.880	89.195	54.334	6.997	421.915	< 0.001
Abnormal oxygen saturation	14.236	6.273	32.309	4.004	1.019	15.729	0.047
Laboratory							
Abnormal leucocytes	17.388	6.056	49.921	5.967	1.162	30.645	0.032
Abnormal pH	18.273	8.437	39.577	11.924	3.583	39.690	< 0.001

;Notes: ^@^ = multiple logistic regression analysis

### The score

To make the final equation easily applicable, a scoring system was derived. The score was calculated based on the coefficients of the variables in the final equation. Some possibilities (of rounding the coefficients) were tried for the score and the best choice was selected based on the area under the ROC curve. Table 6 lists the scoring system. The score also included 6 variables. The performance (sensitivity, specificity, PPV, NPV, LR+, and LR (−)) of the equation and the scoring system are presented in two tables in the Supplementary Material (Additional file 1: 2 and 3). The areas under the ROC Curve for the equation and their score were 95.6 and 95.5%, respectively. The proposed cut off for the equation and the score was 20–40% and 2–3, respectively.

**Table 6:** The Score

Variables	Score
Poorfeeding	• Yes = 2
	• No = 0
Abnormal heart rate (Normal range 100–180 x/minute)	• Yes = 3
	• No = 0
Abnormal temperature (Normal range 36–37.9 °C)	• Yes = 4
	• No = 0
Abnormal oxygen saturation (< 92%)	• Yes = 1
	• No = 0
Abnormal leucocytes ➔ Normal range: < 7 days of age: 9000–30,000 /cmm 7–14 days of age: 5000–21,000 /cmm > 14 days of age: 5000–20,000 /cmm	• Yes = 2
	• No = 0
Abnormal pH (Normal range: 7.27–7.45)	• Yes = 2
	• No = 0

Notes

The maximum score was14

The score equal with the probability in the range:

- Low : 0–2 = 0–20%

- Medium:3–4 = 21–75%

- High :5–6 = 76–95%

- Very High:7–14 = 96–100%

## Discussion

Ninety-seven sepsis patients were identified in this study from 3557 neonatal patients during the 3-year study period. Comparing the incidence of neonatal sepsis in countries was not easy since many reports used different criteria for early- and late-onset neonatal sepsis [42]. In Pakistan, Bosnia, and Malaysia, the incidences of LOS were 29, 71.3, and 90.2%, respectively [1, 23, 43]. Data from four other countries, including Thailand, found an incidence of 5 per 1000 live births [17]. The prevalence was 21.8 or more in Nigeria [12, 13]. A report from the largest hospital in Indonesia found an incidence of 35% [44].

Among all the cases of neonatal sepsis, the percentage of neonates weighing less than 2500 g was 64.1%. Based on the gestational age, the percentage of preterm neonates was 48.9, 69.2, and 59.8% for early-onset sepsis (EOS), LOS, and total sepsis, respectively. These results were similar to other body weight-based reports. Another study reported that the incidence of LOS among very low birth weight (VLBW) neonates was 25–30% and 6–10% in late preterm neonates, with the mortality rate of 36–51% [22]. Data from Kenya and Gambia showed a CFR of 26 and 31% [45, 46].

The percentage of gram-negative organisms in this study was 67.3% (35/52). *Klebsiella pneumoniae* and CONS were the most common microorganisms. These data were comparable with other developing countries [42, 47]. A 10-year prospective surveillance in Brazil revealed 51.6% episodes of neonatal infection caused by gram-negative rods (mainly *Klebsiella* spp. and *E. coli*) [48].

Antibiotics are one of the most important treatments for neonatal sepsis, although some people may not receive this treatment because of the facility limitation in some rural areas [8]. The first line of antibiotics for neonatal sepsis in many countries, like in the studied hospital, are a combination of penicillin group and gentamicin. At least 78% of the LOS patients in this study were administered ampicillin. However, broad-spectrum antibiotics can create problems of resistance. Multi-resistant organisms, such as *A. baumanii* and *K. pneumoniae*, are consistently increasing in many countries, especially in LMIC [8, 44]. Our study focused on bacterial sepsis. All neonatal sepsis patients used antibiotics. This was not used as a decisive variable in our study.

All possible proven neonatal sepsis patients during the 3-year period were included in this study. Nevertheless, this study had a larger sample size than previous studies. The NOSEP Score by Mahieu et al. (2000) used 43 proven episodes and 104 suspected sepsis episodes but did not use non-suspected sepsis patients [49]. Okascharoen et al. (2005) used 1870 neonates, with only 17 proven sepsis patients [16]; Singh et al. (2003) used 30 episodes of definite, 17 most probable, and 58 non-sepsis patients in their study [50]. Recently, the system by Singh was modified using 497 infants in Bangladesh [51]. In 1982, Tollner created the first neonatal sepsis score using basic clinical and laboratory data. He used 667 neonates in Ulm hospital [52].

The dependent variable for this study was proven neonatal sepsis. The proof was mostly based on the culture results, particularly hemoculture. All unproven sepsis patients were excluded. The clearly defined outcome variable is an essential requirement [53]. Confirmed sepsis guaranteed consistency and validity of the outcome [51]. The unproven neonatal sepsis patients were excluded from this study to avoid incorporation bias. This bias would appear if the possible predictive factors became part of the diagnostic criteria [3, 34].

Independent variables in the study originated from previous studies about the predictive model for neonatal sepsis and some scores for neonatal morbidity and mortality. In other clinical prediction rules, predictor variables were identified by the process of selecting, exploring, and modeling large amounts of data to discover unknown patterns or relations [36]. In this study, the independent variables were added by some changes of continuous variables into qualitative forms. Others were made from the unification of some variables.

Initially, the original variables were classified as risk factors / history, clinical conditions, laboratory data, and treatment modalities, as suggested in some previous reports [54]. Some newer laboratory examinations such as procalcitonin [55], various interleukins [56, 57], and PCR methods [58] were not included in this study due to availability and financial reason.

The risk factors included demographic data and maternal history. In this study, the maternal history considered the mother’s habits (smoking, drug use), and the mother’s diseases (fever, amnionitis, history of antibiotics). Maternal diseases significantly contribute to neonatal sepsis—mostly for the early-onset sepsis. Puerperal infection was associated with 2:1 adjusted Risk Ratio for early neonatal mortality. Around 5% of all deaths in the first week of life were attributable to signs suggestive of puerperal infections [59].

To reduce the number of predictor variables and to make the statistical selection, some univariate tests were used as appropriate. In these tests *p* < 0.1 was used, although some other models used *p* < 0.2 [53]. Singh et al. did not use the univariate test for the study [50]. The selection of variables was based on the positive likelihood ratio. The results of the univariate tests were 68 (21 risk factors, 11 clinical condition, 34 laboratories, and 2 treatment modalities) variables.

Multivariate analysis used multiple logistic regression because the outcome variable was dichotomous, and this test was easy [53]. The reselection process was done based on clinical judgment, collinearities (more than 1 variables measured the same thing), similarities, and performances. If continuous and qualitative data were present, the qualitative would be chosen due to practicability. The use of dichotomized data was also accurate and more useful in clinical practice. The original continuous data in NOSEP score derivation did not improve the accuracy of the global scoring system [49].

All the variables were tried one by one several times if more than one choice were available. Gestational age did not pass the univariate test but this variable was tried to enter the multivariate analysis because of its clinical significance [16]. However, this variable still could not be included in the multiple logistic regression results. Some other significant risk factors could not enter the multivariate analysis probably because of the selection of the control group. The choice of non-sepsis neonates would influence the univariate and multivariate results. The final model was selected based on the variable composition, clinical judgment, and performance of the area under the ROC curve [16, 60].

The final equation used 6 variables (4 clinical conditions and 2 laboratory data). Abnormal heart rate had the second-highest adjusted OR after abnormal temperature. Abnormal heart rate characteristics (reduced variability and transient decelerations) occurred early in neonatal sepsis. These abnormalities were present 12–24 h before the clinical diagnosis of sepsis. This method was studied extensively by Griffin et al. in 2001 and 2003 (external validation) [61]. In this study, the normal value was simpler and not calculated using a sophisticated method. Reduced variability and transient decelerations in heart rate may be an early indicator of clinical instability [62, 63].

Abnormal temperature had the highest adjusted OR in the model. This was the most frequent clinical feature in some studies [16, 49]. For term infants, hyperthermia was a high predictive parameter. Some studies showed that more than 50% of the sepsis patients had a fever, while hypothermia was only found among 15% of the infants [64]. In this study, no infant with hypothermia developed late-onset sepsis. This is like the results by Okascharoen et al. (2005). The mortality rate was high among mild and moderate hypothermia in another study and the proportion of hyperthermia and hypothermia was 13 and 13.5%, respectively [65].

Abnormal leucocytes were determined according to Manroe’s criteria [66]. Leucocytes (total white blood cell (WBC) count) are one of the most common tests for evaluating bacterial infections. The criteria by Manroe were still used by some reference books despite its weaknesses, such as depending on the infant’s age, gestational age, and the blood vessels [66, 67]. Abnormal pH—mostly acidosis—would accompany hypoxemia. Metabolic acidosis is, most commonly, a consequence of lactic acid accumulation from anaerobic metabolism in hypoxic infants.

The NOSEP score had 5 final variables (1 risk factor, 1 clinical condition, and 3 laboratory data). The model from Okascharoen et al. had 6 variables (1 risk factor, 3 clinical conditions, and 2 laboratory data), and Singh et al. used 7 final variables (all clinical conditions) [16, 49, 50]. Later, the Hematology Scoring System was revalidated in India using 110 neonates with a good result [68]. Tollner in 1982 used seven clinical parameters, skin color, capillary refill, muscular hypotonia, apnea, respiratory distress, hepatomegaly, and gastrointestinal symptoms [52]. NEO-KISS was a score based on the German national surveillance scoring system. It includes clinical, biochemical, and hematological criteria [69].

Changing the equation into the scoring system will make the usage of the model easier. In comparison with the probability of the equation, the scoring system had a good result. The regression coefficients were used to determine the score [70]. At least 4 possibilities of rounding the coefficients were tried for each group. A different score would produce a different performance of the result. The best system was chosen based on the area under the curve (AUC) of the ROC curve and other performance indicators. The final scoring system for late-onset neonatal sepsis had AUC of 96.6%. The maximum score for this model was 23.

In this study, the AUC was 95.6% for the equation and 95.5% for the score. The sensitivity and specificity of the equation were above 80% for the probability cutoff of 20–40% (equation), or 2–3 (score) However, the choice of cutoff (including the PPV, NPV, LR+, and LR(−)) depends on the purpose of usage. For the balanced sensitivity and specificity, the choice would have to be above 70% of the value.

In the real clinical setting, the score proposes the use of antibiotics for “high” and “very high” groups. In contrast, no antibiotic is required for the “low” group of neonates. For the medium group, the antibiotic decision should be made individually by the attending physician. The clinical prediction rule is not a replacement for clinical judgment and should complement rather than supplant clinical opinion and intuition. Accurate clinical decision making is a central component of patient care [36, 37]. This clinical prediction rule can help the clinician diagnose late-onset neonatal sepsis.

Although some steps in the development were comparable, proper comparison with some other models could not be made easily since each model differs from each other in terms of age criteria, type of variables, validation process, and the purpose of the score. The NOSEP score and Okascharoen’s score use the age criteria of 3 days to determine early- or late-onset sepsis. Rodwell et al. only used the hematology parameter, while Singh et al. (2003) used just clinical conditions [16, 49, 50, 71].

The primary limitation of this study was its retrospective design. Information bias cannot be avoided using that design and data from medical records. The sample size of the study was limited since the total sample had to be divided into 2 groups. The missing data (as an unavoidable part of retrospective design study) was another limitation since any method, however perfect, can lead to biased estimates of the odds ratio and the model performance in predictive models [72]. Regarding the “worst” laboratory results, notably, several biochemistry results might be normal in a septic condition. The choice of patients in the control group (non-sepsis) may also affect the result of the study. For example, in this study, most of the non-sepsis cases had hyperbilirubinemia. The result for the icterus variable might be different if the predominant diagnoses were other diseases. This study also did not use a new data set. However, when our results were compared to the more recent literature, we considered our study to still be appropriate for some settings, especially underdeveloped and developing countries.

The chosen outcome was only proven sepsis. This could result in an underestimation of the true incidence. However, including unproven sepsis would cause incorporation bias. Lastly, validation of a new sample set was needed, either in the same setting or others. It is recommended to prospectively perform the validation process.

## Conclusion

In conclusion, our study developed two predictive models for late-onset neonatal sepsis. One as an equation and another as a scoring system. The predictive models enable clinicians, especially in the resource-limited setting, to have an alternative for microbiological culture. External validation should be done soon to assess the real performance of the other institutions.

## Supplementary information

**Additional file 1: Table S1.** The Coding for the Final Equation. **Table S2.** Performance of The Equation for Bacterial Late-Onset Neonatal Sepsis. **Table S3.**. Performance of The Scoring System for Bacterial Late-Onset Neonatal Sepsis.

## Data Availability

The datasets used and/or analysed during the current study are de-identified and available from the corresponding author on reasonable request.

## Abbreviations

AUC: Area Under the Curve
BUN: Blood Urea Nitrogen
CFR: Case Fatality Rate
CONS: *Coagulase-negative Staphylococcus*
CSF: Cerebrospinal Fluid
ELISA: Enzyme-linked Immunosorbent Assay
EOS: Early Onset Sepsis
ICD: International Classification of Diseases
LMIC: Low and Middle-Income Countries
LOS: Length of Stay
LR: Likelihood Ratio
NICU: Neonatal Intensive Care Unit
NPV: Negative Predictive Value
OR: Odds Ratio
PCR: Polymerase Chain Reaction
PPV: Positive Predictive Value
QSNICH: Queen Sirikit National Institute of Child Health
ROC: Receiver Operating Characteristics
SD: Standard Deviation
SEAMEO: Southeast Asian Ministers of Education Organization
SEM: Standard Error of the Mean
SPSS: Statistical Package for the Social Sciences
TPN: Total Parenteral Nutrition
VLBW: Very Low Birth Weight
WBC: White Blood Cell

## References

[1] Ullah O, Khan A, Ambreen A, Ahmad I, Akhtar T, Gandapor AJ, et al. Antibiotic sensitivity pattern of bacterial isolates of neonatal septicemia in Peshawar**,** Pakistan *Arch Iran Med* 2016;19(12):866–869. 10.1002/sim.4099 27998162

[2] G/eyesus T, Moges F, Eshetie S, Yeshitela B, Abate E. Bacterial etiologic agents causing neonatal sepsis and associated risk factors in Gondar, Northwest Ethiopia**.** BMC Pediatr*.* 2017;17:37.DOI 10.1186/s12887-017-0892-y. 10.1186/s12887-017-0892-y PMC5461759 28587631

[3] Wynn JL (2016). Defining neonatal sepsis. Curr Opin Pediatr.

[4] World Health Organization. Countdown to 2015**,** Maternal, newborn & child survival-fulfilling the health agenda for women and children: the 2014 report. Geneva,WHO Press 2014.

[5] Camacho-Gonzalez A, Spearman PW, Stoll BJ (2013). Neonatal infectious diseases: evaluation of neonatal sepsis. Pediatr Clin N Am.

[6] Liu L, Johnson HL, Cousens S, Perin J, Scott S, Lawn JE (2012). Global regional, and national causes of child mortality: an updated systematic analysis for 2010 with time trends since 2000. Lancet.

[7] Sankar MJ, Natarajan CK, Das RR, Agarwal R, Chandrasekaran A, Paul VK (2016). When do newborns die? A systematic review of timing of overall and cause-specific neonatal deaths in developing countries. J Perinatol.

[8] Waters D, Jawad I, Ahmad A, Luksic I, Nair H, Zgaga L (2011). Aetiology of community acquired neonatal sepsis in low- and middle-income countries. J Global Health.

[9] Amare D, Mela M, Dessie G (2019). Unfinished agenda of the neonates in developing countries: magnitude of neonatal sepsis: systematic review and meta-analysis. Heliyon.

[10] Sankar MJ, Chaurasia S, Sivanandan S, Agarwal R, Ellis S, Sharland M (2019). Neonatal sepsis in South Asia: huge burden and spiralling antimicrobial resistance. Br Med J.

[11] Murthy S, Godinho MA, Guddattu V, Edward L, Lewis S, Nair NS (2019). Risk factors of neonatal sepsis in India: a systematic review and meta-analysis. PLoS One.

[12] Arowosegbe AO, Ojo DA, Dedeke IO, Shittu OB, Akingbade OA (2017). Neonatal sepsis in a Nigerian tertiary hospital: clinical features, clinical outcome, aetiology, and antibiotic susceptibility pattern. South Africa J Infect Dis.

[13] Peterside O, Pondei K, Akinbami FO (2015). Bacteriological profile and antibiotic susceptibility pattern of neonatal sepsis at a teaching hospital in Bayelsa state. Nigeria Trop Med Health.

[14] Hasibuan B (ed). Comparison of microbial pattern in early and late onset neonatal sepsis in referral center Haji Adam Malik Hospital Medan Indonesia. *IOP Conference Series: Earth and environmental science*. IOP Publishing 2018.

[15] Yossuck P, Predisripipat K (2002). Neonatal GBS infection: incidence and clinical manifestation in Siriraj hospital. J Med Assoc Thail.

[16] Okascharoen C, Sirinavin S, Thakkinstian A, Kitayaporn D, Supapanachart S (2005). A bedside prediction scoring model for late-onset neonatal sepsis. J Perinatol.

[17] Al Taiar A, Hammoud MS, Thalib L, Isaacs D (2011). Pattern and etiology of culture-proven early-onset neonatal sepsis: a five-year prospective study. Int J Infect Dis.

[18] Paolucci M, Landini MP, Sambri V. How can the microbiologist help in diagnosing neonatal sepsis ? *Int J Pediatr.* 2012;ID 120139;doi:10.1155/2012/120139. 10.1155/2012/120139 PMC3272815 22319539

[19] Satar M, Ozlu F (2012). Neonatal sepsis: a continuing disease burden. Turk J Pediatr.

[20] Polin RA (2012). Committee on fetus and newborn. Management of neonates with suspected or proven early-onset bacterial sepsis. Pediatrics.

[21] Zhou B, Liu X, Wu JB, Jin B, Zhang YY (2016). Clinical and microbiological profile of babies born with risk of neonatal sepsis. Exp Ther Med.

[22] Cortese F, Scicchitano P, Gesualdo M, Filaninno A, De Giorgi E, Schettini F (2016). Early and late infections in newborns: where do we stand?. A review Pediatr Neonatol.

[23] Softic I, Tahirovic H, Di Ciommo V, Auriti C (2017). Bacterial sepsis in neonates: single center study in a neonatal intensive care unit in Bosnia and Herzegovina. Acta Med Acad.

[24] Hoffmann MA, Snowden JN, Simonsen KA, Nenninger TM, Lyden ER, Al A-B (2015). Neonatal late-onset sepsis following peripherally inserted central catheter removal: association with antibiotic use and adverse line events. J Infus Nurs.

[25] Hornik CP, Fort P, Clark RH, Watt K, Benjamin DK, Smith PB (2014). Early and late-onset sepsis in very low birth weight infants from a large group of neonatal intensive care units. Early Hum Dev.

[26] Stoll BJ, Hansen NI, Bell EF, Laptook AR, Walsh MC, Hale EC (2010). Neonatal outcomes of extremely preterm infants from the NICHD neonatal research network. Pediatrics.

[27] Gebremedhin D, Berhe H, Gebrekirstos K (2016). Risk factors for neonatal sepsis in public hospitals of Mekelle City, North Ethiopia, unmatched case-control study. PLoS One.

[28] Sarafidis K, Chatziioannou AC, Thomaidou A, Gika H, Mikros E, Benaki D (2017). Urine metabolomics in neonates with late-onset sepsis in a case-control study. Sci Rep.

[29] Mishra UK, Jacobs SE, Doyle LW, Garland SM (2006). Newer approaches to the diagnosis of early onset neonatal sepsis. Arch Dis Child Fetal Neonatal Ed.

[30] Rozanska A, Bulanda M (2015). Infections and risk-adjusted length of stay and hospital mortality in polish neonatology intensive care units. Int J Infect Dis.

[31] The Delhi Neonatal Infection Study (DeNIS) Collaboration (2016). Characterisation and antimicrobial resistance of sepsis pathogens in neonates born in tertiary care centers in Delhi, India: a cohort study. Lancet Glob Health.

[32] Ng PC, Lam HS (2012). Biomarkers in neonatology: the next generation of tests. Neonatology.

[33] Verstraete EH, Blot K, Mahieu L, Vogelaers D, Blot S (2015). Prediction models for neonatal healthcare-associated sepsis: a meta-analysis. Pediatrics.

[34] Khaertynov KS, Boichuk SV, Khaiboullina SF, Anokhin VA, Andreeva AA, Lombardi VC, et al. Comparative assessment of cytokine pattern in early and late onset of neonatal sepsis. *J Immunol Res.* 2017;ID 8601063: 10.1155/2017/8601063. 10.1155/2017/8601063 PMC5357566 28367457

[35] Shah B, Padbury JF (2014). Neonatal sepsis. An old problem with new insights. Virulence.

[36] Adam ST, Leveson SH (2012). Clinical prediction rules. Br Med J.

[37] Laupacis A, Sekar N, Stiell IG (1997). Clinical prediction rules. J Am Med Assoc.

[38] Stanton TR (2016). Clinical prediction rules that Don't hold up—where to go from Here?. J Ortho Sports Phys Ther.

[39] Reilly BM, Evans AT (2006). Translating clinical research into clinical practice: impact of using prediction rules to make decisions. Ann Intern Med.

[40] McGinn TG, Guyatt GH, Wyer PC, Naylor CD, Stiell IG, Richardson WS (2000). Users’ guide to the medical literature; how to use articles about clinical decision rules. J Am Med Assoc.

[41] Sanders S, Doust J, Glasziou P (2015). A systematic review of studies comparing diagnostic clinical prediction rules with clinical judgment. PLoS One.

[42] Vergnano S, Sharland M, Kazembe P, Mwansambo C, Heath PT (2005). Neonatal sepsis: an international perspective. Arch Dis Child Fetal Neonatal Ed.

[43] Boo NY, Cheah IGS. Factors associated with inter-institutional variations in sepsis rates of very low birth weight infants in 34 Malaysian neonatal intensive care units. *Singapore Med J.* 2016;57(3):144–52.doi:10.11622/smedj.2016056 10.11622/smedj.2016056 PMC4800725 26996633

[44] Tjoa E, Moehario LH, Rukmana A, Rohsiswatmo R. *Acinetobacter baumanii*: role in blood stream infection in neonatal unit Dr. Cipto Mangunkusumo Hospital, Jakarta, Indonesia. *Int J Microbiol*.2013: ID180763. 10.1155/2013/180763 PMC3830835 24288538

[45] Berkley JA, Lowe BS, Mwangi I, Williams T, Bauni E, Mwarumba S (2005). Bacteremia among children admitted to a rural hospital in Kenya. Lancet.

[46] Mulholland K, Ogunlesi O, Adegbola R, Weber M, Sam B, Palmer A (1999). Etiology of serious infections in young Gambian infants. Pediatr Infect Dis J.

[47] Ghotaslou R, Ghorashi Z, Nahaei MR. *Klebsiella pneumoniae* in neonatal sepsis: a 3 year study in the pediatric hospital of Tabriz**,** Iran *Jpn J Infect Dis* 2007;60:126–128. 17515647

[48] Couto RC, Carvalho EAA, Pedrosa TMG, Pedroso ER, Neto MC, Biscione FM (2007). A 10-year prospective surveillance of nosocomial infections in neonatal intensive care units. Am J Infect Control.

[49] Mahieu LM, De Muynck AO, De Dooy JJ (2000). Prediction of nosocomial sepsis in neonates by means of a computer-weighted bedside scoring system (NOSEP score). Crit Care Med.

[50] Singh SA, Dutta S, Narang A (2003). Predictive clinical scores for diagnosis of late-onset neonatal septicemia. J Trop Pediatr.

[51] Rosenberg RE, Ahmed ASMNU, Saha SK, Chowdhury MAKA, Ahmed S, Law PA (2010). Nosocomial sepsis risk score for preterm infants in low resource settings. J Trop Pediatr.

[52] Tollner U (1982). Early diagnosis of septicemia in the newborn. Clinical studies and sepsis score. Eur J Pediatr.

[53] Guyatt G. Determining prognosis and creating clinical decision rules. In: Haynes RB, Sackett DL, Guyatt GH, Tugwell P. *Clinical epidemiology. Third edition.* Philadelphia, Lippincott Williams & Wilkins 2006,323–55.

[54] Smith PB, Benjamin DK, Long SS, Pickering LK, Prober CG (2008). Clinical approach to the infected neonate. Principles and practices of pediatric infectious diseases.

[55] Pontrelli G, De Crescenzo F, Buzzetti R, Jenkner A, Balduzzi S, Carducci FC, et al. Accuracy of serum procalcitonin for the diagnosis of sepsis in neonates and children with systemic inflammatory syndrome: a meta-analysis. *BMC Infect Dis.* 2017;17:302.DOI 10.1186/s12879-017-2396-7. 10.1186/s12879-017-2396-7 PMC5404674 28438138

[56] Wagner TA, Gravett CA, Healy S, Soma V, Patterson JC, Gravett MG (2011). Emerging biomarkers for the diagnosis of severe neonatal infections applicable to low-resource settings. J Glob Health.

[57] Meem M, Modak JK, Mortuza R, Morshed M, Islam MS, Saha SK (2011). Biomarkers for diagnosis of neonatal infections: a systematic analysis of their potential as a point of care diagnostics. J Glob Health.

[58] Ng PC, Lam HS (2010). Biomarkers for late-onset neonatal sepsis: cytokines and beyond. Clin Perinatol.

[59] Bellizzi S, Bassat Q, Ali MM, Sobel HL, Temmerman M (2017). Effect of puerperal infections on early neonatal mortality: a secondary analysis of six demographic and health surveys. PLoS One.

[60] Toll DB, Janssen KJ, Vergouwe Y, Moons KG (2008). Validation, updating and impact of clinical prediction rules: a review. J Clin Epidemiol.

[61] Griffin MP, Lake DE, Moorman JE (2005). Heart rate characteristics and laboratory tests in neonatal sepsis. Pediatrics..

[62] Moorman JR, Carlo WA, Kattwinkel J, Schelonka RL, Porcelli PJ, Navarette CT (2011). Mortality reduction by heart rate characteristics monitoring in very low birth weight neonates: a randomized trial. J Pediatr.

[63] Bhatti M, Chu A, Hageman JR, Schreiber M, Alexander K (2012). Future directions in the evaluation and management of neonatal sepsis. Neoreviews.

[64] Estripeaut D, Saez-Llorenz X, Feigin RD, Cherry JD, Demmler-Harrison GJ, Kaplan SL (2009). Perinatal bacterial diseases. Feigin & Cherry’s textbook of pediatric infectious diseases.

[65] Ahmad MS, Ali N, Mehboob N, Mehmood R, Ahmad M, Wahid A (2016). Temperature on admission among cases of neonatal sepsis and its association with mortality. J Pak Med Assoc.

[66] Manroe BL, Weinberg AG, Rosenfeld CR, Browne R (1979). The neonatal blood count in health and disease. I. Reference values for neutrophilic cells. J Pediatr.

[67] Schelonka RL, Yoder BA, desJardins SE, hall RB, Butler J. peripheral leucocyte count and leucocyte indices in healthy newborn infants. J Pediatr 1994;125:603–606. 10.1016/s0022-3476(94)70018-4 7931882

[68] Makkar M, Gupta C, Pathak R, Garg S, Mahajan NC (2013). Performance evaluation of hematologic scoring system in early diagnosis of neonatal sepsis. J Clin Neonatol.

[69] Gastmeier P, Geffers C, Schwab F, Fitzner J, Obladen M, Ruden H (2004). Development of a surveillance system for nosocomial infections: the component for neonatal intensive care units in Germany. J Hosp Infect.

[70] Moons KGM, Donders RART, Stijnen T, Harrell FE (2006). Using the outcome for imputation of missing predictor values was preferred. J Clin Epidemiol.

[71] Rodwell RL, Leslie AL, Tudehope DI (1988). Early diagnosis of neonatal sepsis using a hematologic scoring system. J Pediatr.

[72] Gorelick MH (2006). Bias arising from missing data in predictive models. J Clin Epidemiol.

