# Supplementary material for: Predictive model for bacterial late-onset neonatal sepsis in a tertiary care hospital in Thailand
Source: BMC Infect Dis. 2020 Feb 18;20:151. doi: 10.1186/s12879-020-4875-5 (PMC7029566; doi:10.1186/s12879-020-4875-5)
Supplement: Supplementary file 1 — Additional file 1: Table S1. The Coding for the Final Equation. Table S2. Performance of The Equation for Bacterial Late-Onset Neonatal Sepsis. Table S3.. Performance of The Scoring System for Bacterial Late-Onset Neonatal Sepsis. [file 12879_2020_4875_MOESM1_ESM.docx]

**Supplementary Material 1**

**The Coding for the Final Equation**

| **VARIABLES** | **CODING** |
| --- | --- |
| Poor feeding  Abnormal heart rate ( Normal range :  100 – 180 x/minute )  Abnormal temperature ( Normal range :  36 – 37.9^o^C )  Abnormal oxygen saturation ( < 92% )  Abnormal leucocytes 🡪 Normal range :  < 7 days of age : 9000 – 30 000 /cmm  7-14 days of age : 5000 – 21 000 /cmm  > 14 days of age : 5000 – 20 000 /cmm  Abnormal pH ( Normal range : 7.27 – 7.45 ) | - Yes = 1 - No = 0 - Yes = 1 - No = 0 - Yes = 1 - No = 0 - Yes = 1 - No = 0 - Yes = 1 - No = 0 - Yes = 1 - No = 0 |

**Supplementary Material 2**

**Performance of The Equation for Bacterial Late-Onset Neonatal Sepsis**

| **PROBABILITY**  **CUTOFF^#^ ( % )** | **EQUATION** | |
| --- | --- | --- |
|  | **SENSITIVITY** | **SPECIFICITY** |
| **0**  **10.00**  **20.00**  **30.00**  **40.00**  **50.00** | 100  94.2  86.6  86.6  80.8  73.1 | 0  73.7  90.4  91.7  93.6  96.2 |

Notes: ^#^ = cutoff of the diagnosis of bacterial late-onset neonatal sepsis

**Supplementary Material 3**

**Performance of The Scoring System for Bacterial Late-Onset Neonatal Sepsis**

| **CUTOFF^#^**  **( TOTAL SCORE )** | **SCORE SYSTEM** | | | | | |
| --- | --- | --- | --- | --- | --- | --- |
|  | **SENSITI**  **VITY** | **SPECIFI**  **CITY** | **PPV** | **NPV** | **LR+** | **LR-** |
| 0  1  2  3  4  5 | 98.1  96.2  88.5  82.7  65.4  59.6 | 69.9  72.4  90.4  93.6  98.7  100 | 52  53.8  75.4  81.1  94.4  100 | 99.1  98.3  95.9  94.2  89.5  88.1 | 3.26  3.49  9.22  12.92  50.31 | 0.03  0.05  0.13  0.19  0.35  0.40 |

Notes: PPV = positive predictive value; NPV = negative predictive value ; LR+ = positive likelihood ratio ; LR- = negative likelihood ratio ; ^#^ = cutoff of the diagnosis of bacterial late-onset neonatal sepsis
